# Identification of Mitochondrial DNA Polymorphisms That Alter Mitochondrial Matrix pH and Intracellular Calcium Dynamics

**DOI:** 10.1371/journal.pgen.0020128

**Published:** 2006-08-11

**Authors:** An-a Kazuno, Kae Munakata, Takeharu Nagai, Satoshi Shimozono, Masashi Tanaka, Makoto Yoneda, Nobumasa Kato, Atsushi Miyawaki, Tadafumi Kato

**Affiliations:** 1 Laboratory for Molecular Dynamics of Mental Disorders, Brain Science Institute, RIKEN, Saitama, Japan; 2 Department of Neuropsychiatry, Faculty of Medicine, University of Tokyo, Tokyo, Japan; 3 Laboratory for Cell Function and Dynamics, Brain Science Institute, RIKEN, Saitama, Japan; 4 Structure and Function of Biomolecules, Precursory Research for Embryonic Science and Technology, Japan Science and Technology Agency, Saitama, Japan; 5 Genomics for Longevity and Health, Tokyo Metropolitan Institute of Gerontology, Tokyo, Japan; 6 Second Department of Internal Medicine, University of Fukui Faculty of Medical Sciences, Fukui, Japan; University of Minnesota, United States of America

## Abstract

Mitochondrial DNA (mtDNA) is highly polymorphic, and its variations in humans may contribute to individual differences in function as well as susceptibility to various diseases such as Parkinson disease, Alzheimer disease, bipolar disorder, and cancer. However, it is unclear whether and how mtDNA polymorphisms affect intracellular function, such as calcium signaling or pH regulation. Here we searched for mtDNA polymorphisms that have intracellular functional significance using transmitochondrial hybrid cells (cybrids) carrying ratiometric Pericam (RP), a fluorescent calcium indicator, targeted to the mitochondria and nucleus. By analyzing the entire mtDNA sequence in 35 cybrid lines, we found that two closely linked nonsynonymous polymorphisms, *8701A* and *10398A,* increased the basal fluorescence ratio of mitochondria-targeted RP. Mitochondrial matrix pH was lower in the cybrids with *8701A*/*10398A* than it was in those with *8701G*/*10398G,* suggesting that the difference observed by RP was mainly caused by alterations in mitochondrial calcium levels. Cytosolic calcium response to histamine also tended to be higher in the cybrids with *8701A*/*10398A.* It has previously been reported that *10398A* is associated with an increased risk of Parkinson disease, Alzheimer disease, bipolar disorder, and cancer, whereas *10398G* associates with longevity. Our findings suggest that these mtDNA polymorphisms may play a role in the pathophysiology of these complex diseases by affecting mitochondrial matrix pH and intracellular calcium dynamics.

## Synopsis

Mitochondria play important roles in energy production and regulation of intracellular calcium levels. Mitochondria have their own genetic material, mitochondrial DNA (mtDNA). In spite of its short length (16 kbp), mtDNA is highly variable among individuals and is thought to contribute to interindividual functional variability in energy-requiring activities such as intelligence and athletic performance. However, it is unclear whether mtDNA polymorphisms affect intracellular function and condition. Using transmitochondrial hybrid cells, the authors found two closely linked mtDNA polymorphisms, *10398A*/*G* and *8701A*/*G,* which cause alterations in mitochondrial pH and calcium concentration. Cytosolic calcium response to histamine tended to be different between transmitochondrial hybrid cells carrying these two mtDNA polymorphisms. It has been reported that the *10398A* mtDNA polymorphism is a risk factor for Parkinson disease, Alzheimer disease, cancer, and bipolar disorder, whereas *10398G* is associated with longevity. The present findings suggest that these mtDNA polymorphisms may play a role in the pathophysiology of these complex diseases by affecting mitochondrial matrix pH and intracellular calcium dynamics.

## Introduction

The central importance of mitochondria in ATP production is well established [1,2]. The pH gradient across the mitochondrial membrane and the inner mitochondrial membrane potential make up the electrochemical gradient, which regulates the efficiency of ATP synthesis and other mitochondrial activity. Recent studies are also focusing on the roles of mitochondria in regulation of intracellular calcium dynamics. Mitochondrial calcium uptake affects various important cellular processes such as apoptosis [3–5], exocytosis [6,7], synaptic plasticity [8], and possibly spine dynamics [9].

Mitochondria have their own DNA, mitochondrial DNA (mtDNA), which encodes the genes of 22 transfer RNAs (tRNAs), 2 ribosomal RNAs (rRNAs), and 13 subunits of enzymes related to oxidative phosphorylation [10]. Other subunits of mitochondrial proteins are encoded in the nuclear genome.

Mutations in mtDNA are known to cause various mitochondrial diseases such as mitochondrial myopathy, encephalopathy, lactic acidosis, and stroke-like episodes (MELAS), which is caused by the *3243A/G* mutation in mtDNA [11,12]. The mechanisms by which these mtDNA mutations cause functional impairment are well studied [13,14]. On the other hand, mtDNA is highly polymorphic, and certain polymorphisms are thought to be risk factors in complex diseases such as diabetes mellitus [15], Alzheimer disease [16,17], Parkinson disease [18–21], bipolar disorder [22,23], and some kinds of cancer [24,25]. It has also been reported that mtDNA polymorphisms are related to interindividual functional variability in human cognition [26], personality [27], athletic performance [28], and longevity [29]. However, these associations are solely dependent on population genetics. It is difficult to draw a definite conclusion from genetic association analyses alone because the high variability of mtDNA among individuals makes such analysis susceptible to confounding effects of population stratification; in addition, the effects of other polymorphisms in mitochondrial or nuclear genes are difficult to control. Although functional analyses of these polymorphisms are needed, to date there are few reports that identify functional effects of mtDNA polymorphisms. This is mainly due to methodological difficulties, as conventional molecular biological techniques are not readily applicable to mtDNA.

We examined the phenotypic effect of mitochondrial DNA without interference from nuclear genes by analysing transmitochondrial hybrid cells (cybrids), which were made by fusing a cell line lacking mtDNA, called ρ^0^ (rho zero) cells [30], with platelets from humans. Although the consequences of pathogenic mtDNA mutations have been examined using this technique, the functional significance of mtDNA polymorphisms has not been well investigated yet.

In this study, we searched for functional mtDNA polymorphisms using the following strategies: (1) by targeting a calcium indicator, ratiometric Pericam (RP) [31], to mitochondria and the nucleus in the same cell, mitochondrial and cytosolic calcium levels were monitored simultaneously; (2) to reduce cellular variability, a ρ^0^ cell line was subcloned before generation of cybrids; (3) by sequencing the whole mtDNA genome in 35 cybrids, functional mtDNA polymorphisms were comprehensively analyzed, and two nonsynonymous mtDNA polymorphisms, *10398A*/*G* and *8701A*/*G,* were identified; and (4) using a pH indicator, we confirmed that these mtDNA polymorphisms altered both mitochondrial matrix pH and intracellular calcium levels.

## Results

### Generation and Confirmation of Cybrid Cell Lines

First, we established a 143B.TK^−^ ρ^0^206 cell line that stably expresses two RPs. RP is a ratiometric fluorescent protein developed as a calcium indicator [31]. The fluorescence ratio of 510 nm emission at 480 nm excitation to that at 410 nm excitation is reported to reflect calcium concentration at a constant pH. Mitochondria-targeted RP is also sensitive to pH because there is a higher pH [32,33] in the mitochondrial matrix (approximately 7.7–8.0) [34,35] than in the cytosol. In this study, two RPs, one targeted to mitochondria (mtRP) and the other to the nucleus (nucRP), were expressed in the same living cell. Thus, we were able to simultaneously monitor mitochondrial and cytosolic calcium concentrations [31]. Because calcium levels in the nucleus were reported to be similar to those in cytosol, nuclear calcium levels were used to indicate cytosolic calcium level.

We subcloned several ρ^0^ cell lines carrying RPs and selected one that showed a reproducible calcium response. We confirmed by mtDNA-specific PCR and Southern blot analysis that this cell line lacked mtDNA. We fused this cell line with platelets taken from 35 human volunteers and confirmed the integration of mtDNA from the volunteers by Southern blot analysis.

### Measurements of Fluorescence Ratio of mtRP in all Cybrid Cell Lines

**Figure 1:**
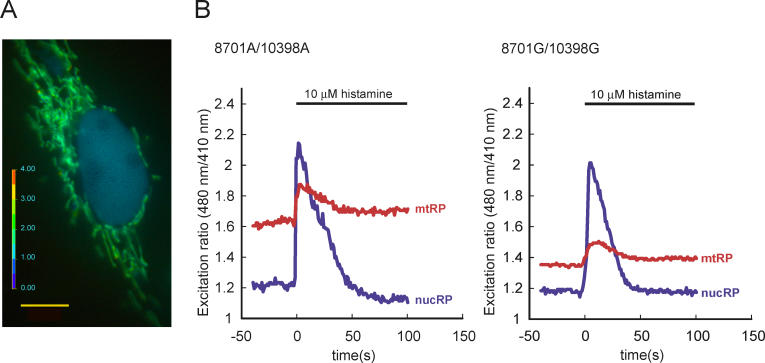
Typical Excitation Ratio Image of a Cybrid and Histograms Showing Responses to Histamine (A) Image of a typical cybrid showing fluorescence ratios of excitation at 480 nm to 410 nm in pseudocolors. Mitochondria had a higher 480 nm/410 nm ratio than the nucleus, possibly reflecting higher calcium levels and pH in mitochondrial matrix than in cytosol. Scale bar, 10 μm. (B) Representative fluorescence ratio responses of *8701A*/*10398A* cybrids (left) and *8701G*/*10398G* cybrids (right) evoked by 10 μM histamine. The *y* axis shows the ratio of the 525 nm fluorescence at 480 nm to that at 410 nm. Blue traces show the ratio in the nucleus and red traces show the ratio in mitochondria. Histamine (10 μM) was added at time 0 s and was not washed out during the experiment.

Using these cybrids, we measured fluorescence ratios of mtRP and nucRP. In fluorescent images of cybrids, mitochondria had a higher 480 nm/410 nm ratio than the nucleus (Figure 1A), possibly reflecting higher calcium levels and pH in mitochondrial matrix than in cytosol. This result is consistent with previous observations in HeLa cells [32]. At first, baseline fluorescence ratios were recorded for 2 minutes. Subsequently, cells were stimulated by 10 μM histamine, which elicits inositol trisphosphate–mediated calcium release from the endoplasmic reticulum [36]. In the first 60 s following stimulation, the fluorescence ratio of mtRP and nucRP initially increased and then returned to basal levels (Figure 1B). The mtRP fluorescence ratio was measured in 11–42 cells for each of the 35 cell lines by fluorescent microscopy.

Using a one-way analysis of variance, we tested whether or not interindividual variation of mtDNA causes differences in basal mtRP fluorescence ratios. A statistically significant variation of basal mtRP fluorescence ratios was found among these cybrid lines (*p* < 0.001, *F* = 6.328).

### Entire Sequences of mtDNA in All Cybrid Cell Lines

To identify the mtDNA polymorphisms that cause heterogeneity among cybrids, we determined the entire 16.5-kbp sequence of mtDNA in each of the 35 cybrids. mtDNA polymorphisms were selected in comparison with the revised Cambridge Reference Sequence [10,37]. A total of 216 polymorphic sites, including 13 novel polymorphisms, were identified. All cybrids had different sequences.

### Identification of mtDNA Polymorphisms Altering the Fluorescence Ratio of mtRP

To identify the polymorphisms with functional significance, all nonsynonymous polymorphisms or polymorphisms in tRNA or rRNA found in more than three cybrids (*n* = 16) were selected (Table 1). For each polymorphism, the average mtRP fluorescence ratios for two genotypes at the particular polymorphic site were compared (Table 1). The polymorphisms at only two positions, 8701 and 10398, showed nominally significant differences of basal mtRP fluorescence ratios. Basal mtRP fluorescence ratios in cybrids with mtDNA *10398A* were significantly higher than those with *10398G* (*10398A,* 1.67 ± 0.09 [mean ± SD], *n* = 11; *10398G,* 1.59 ± 0.12 [mean ± SD], *n* = 24; *p* < 0.05, Mann-Whitney *U* test). Basal mtRP fluorescence ratios in the cybrids with *8701A* were also significantly higher than those with *8701G* (*8701A,* 1.67 ± 0.1 [mean ± SD], *n* = 13; *8701G,* 1.59 ± 0.11 [mean ± SD], *n* = 22; *p* < 0.05). Because *8701A* was linked to *10398A* in all but two cybrids, it was difficult to assess the independent effects of these polymorphisms. Since these two cybrids showed intermediate values (average fluorescence ratio, 1.64), it suggests that both of these polymorphisms may contribute to the observed difference. Because there were only two subjects with *8701G*/*10398A,* further analyses were performed using only *8701A*/*10398A* and *8701G*/*10398G* cybrids.

**Table 1:**
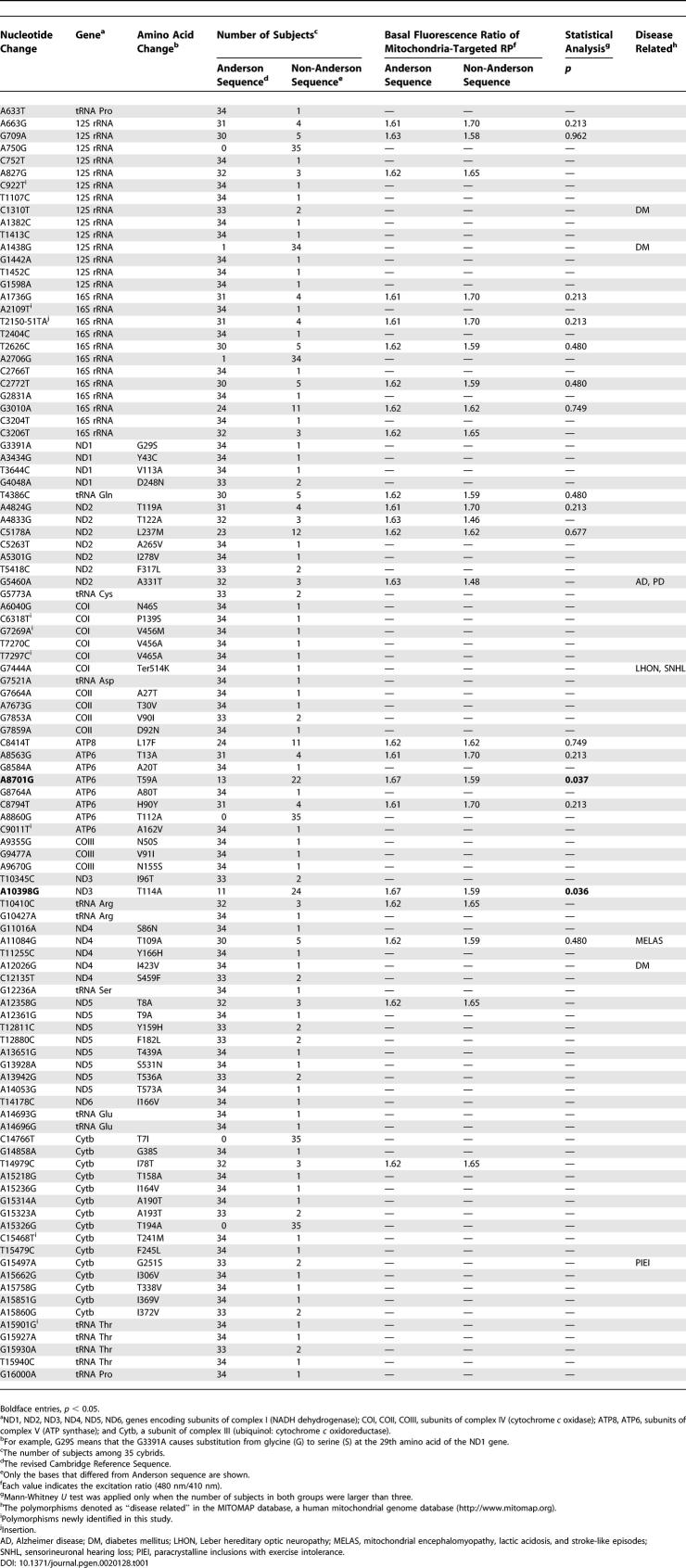
The mtDNA Polymorphisms in 35 Cybrids

**Figure 2:**
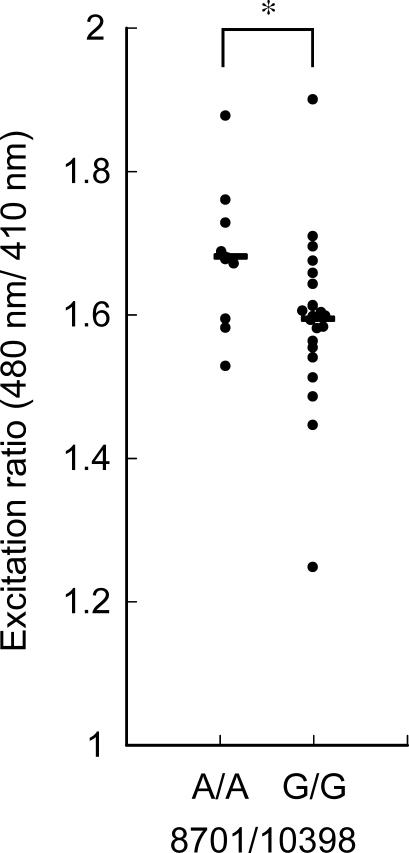
Effects of *10398A*/*G* on Basal Fluorescence Ratios of Mitochondria-Targeted RP Differences between the basal fluorescence ratios in *10398A* and *10398G* cybrids are shown. All cybrids with *8701A* have *10398A* (left). While most cybrids with *8701G* had *10398G* (right), two cybrids had *8701A* and *10398G* (the data of these two cybrids were 1.52 and 1.77). For each cybrid, data taken from 11–42 cells (average, 26 cells) were averaged. The *y* axis shows the ratio of the 525 nm fluorescence at 480 nm to that at 410 nm. Horizontal bars indicate the mean values for each type of cybrid. *p < 0.05 (Mann-Whitney *U* test).

Basal mtRP fluorescence ratios in cybrids with *8701A*/*10398A* mtDNA were significantly higher than those with *8701G*/*10398G* mtDNA (*8701A*/*10398A,* 1.67 ± 0.09 [mean ± SD], *n* = 11; *8701G*/*10398G,* 1.59 ± 0.11 [mean ± SD], *n* = 22; *p* < 0.05, Mann-Whitney *U* test; Figures 1 and 2). Peak mtRP fluorescence ratios after the histamine stimulation also showed a similar but nonsignificant trend (*8701A*/*10398A,* 1.86 ± 0.11 [mean ± SD]; *8701G*/*10398G,* 1.78 ± 0.14 [mean ± SD]; *p* = 0.05).

### Replication Study Using Cybrid Cell Lines Stably Expressing RP

Because the nominally significant effects of polymorphisms at positions 10398 and 8701 on basal mtRP fluorescence ratio were found after multiple statistical analyses, we performed a second study to ensure it was not a false-positive finding. Three cybrid lines with *8701A*/*10398A* mtDNA and three with *8701G*/*10398G* mtDNA were chosen from among the 35 cybrids for the replication study (Table S1). The results confirmed our initial observation of a higher basal mtRP fluorescence ratio in cybrids with mtDNA *8701A*/*10398A* (*8701A*/*10398A,* 1.78 ± 0.04 [mean ± SD], three cell lines; *8701G*/*10398G,* 1.67 ± 0.01 [mean ± SD], three cell lines). Peak mtRP fluorescence ratios after the histamine stimulation also showed a similar trend (*8701A*/*10398A,* 1.99 ± 0.04 [mean ± SD], three cell lines; *8701G*/*10398G,* 1.88 ± 0.02 [mean ± SD], three cell lines).

### Replication Study Using Independently Established Cybrid Cell Lines

To further confirm that the observed difference is caused by the difference of mtDNA sequence, we performed another replication study using independently established cybrid cell lines. In this experiment, the native 143B.TK^−^ ρ^0^206 cell line was used without any further subcloning. We established independent cybrid cell lines by using platelets derived from a subgroup of the original 35 donors (Table S1). By transiently transfecting the cybrids with *ratiometric coxIV-Pericam* cDNA, fluorescence imaging was performed. Moreover, cells were stimulated by 10 μM histamine, and in vivo calibration was performed using calibration buffers with ionomycin, which equilibrate intramitochondrial and extracellular calcium. To minimize the cell-to-cell variability, an index for calcium level, *(R* − *R*_min_ *) / (R*_max_ − *R)* (see Materials and Methods), was analyzed in cells responded to histamine. As a result, our initial observation in cybrids with mtDNA *8701A*/*10398A* was confirmed (*8701A*/*10398A,* 1.07 ± 0.40 [mean ± SD], three cell lines; *8701G*/*10398G,* 0.51 ± 0.09 [mean ± SD], three cell lines; Figure S1).

### Generation of pH Indicator for Measurements of Mitochondrial Matrix pH

It has been reported that the fluorescence ratio of RP is dependent not only on calcium levels but also on pH [32,33], especially at the higher pH range found in the mitochondrial matrix. To test the relative contributions of these factors to the observed finding, we attempted to measure mitochondrial pH using mitochondria-targeted, pH-sensitive green fluorescent protein (mt pH-GFP) [38,39] and mitochondria-targeted red fluorescent protein from *Discosoma* (mt DsRed) expressed under the control of a bidirectional promoter [40–42]. Because the pKa of pH-GFP is approximately 8.0, it is useful for measuring mitochondrial matrix pH. Since DsRed is a pH-insensitive protein, it can be used as a reference [43].

We confirmed that this pH indicator, mt pH-GFP/DsRed, could be successfully used to measure mitochondrial pH.

### Measurements of Mitochondrial Matrix pH in Cybrids with mtDNA *8701A*/*10398A* and *8701G*/*10398G*

**Figure 3:**
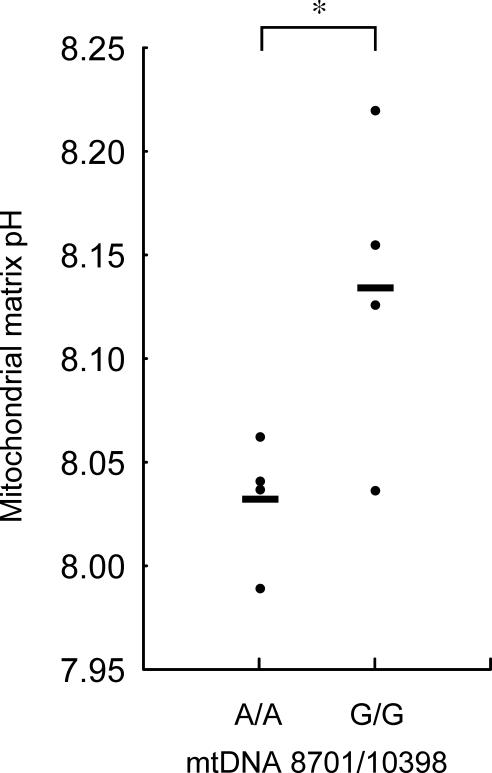
Basal Mitochondrial Matrix pH in the Cybrids with *8701A*/*10398A* or *8701G*/*10398G* Data from 11–18 cells in a triplicate experiment were averaged for each of four cell lines in each group. The *y* axis shows mitochondrial matrix pH calculated by calibration in vivo. Horizontal bars indicate the mean values for each type of cybrid. *p < 0.05 (*t* test).

For the measurements of mitochondrial matrix pH, we used the above-mentioned independently established lines of cybrids generated from the native 143B.TK^−^ ρ^0^ 206 cell line that does not carry RPs (four with *8701A*/*10398A* and four with *8701G*/*10398G*). By transiently cotransfecting the mt pH-GFP/DsRed and Tet-Off vectors, basal mitochondrial matrix pH was recorded for several minutes in each cybrid cell line. We performed in vivo calibration of mitochondrial basal matrix pH in each cell by using four calibration buffers (pH 7.0, 7.5, 8.0, and 8.5) with the ionophores nigericin and monensin, which equilibrate intramitochondrial and extracellular pH. The calculated basal mitochondrial matrix pH was about 8.0, which is consistent with previous reports using HeLa cells and ECV304 cells [34,35]. Compared with the *8701G*/*10398G* cybrids, the basal mitochondrial matrix pH was significantly lower in the *8701A*/*10398A* cybrids (*8701A*/*10398A,* 8.03 ± 0.03 [mean ± SD], four cell lines; *8701G*/*10398G,* 8.13 ± 0.07 [mean ± SD], four cell lines; *p* < 0.05, *t* test; Figure 3).

### Cytosolic Calcium Dynamics

To test whether or not these mtDNA polymorphisms affect cytosolic calcium dynamics, we examined the effects of *8701A*/*10398A* and *8701G*/*10398G* genotypes on the cytosolic calcium levels.

**Figure 4:**
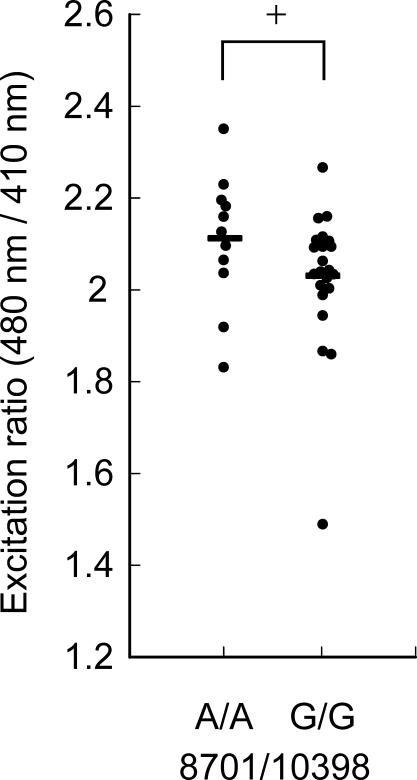
Peak Cytosolic Calcium Level after Histamine Stimulation in Cybrids with *8701A*/*10398A* and *8701G*/*10398G* The values of two cybrids with *8701A*/*10398G* were 1.92 and 2.20. For each cybrid, data taken from 11–42 cells (average, 26 cells) were averaged. The *y* axis shows the ratio of the 525 nm fluorescence at 480 nm to that at 410 nm. Horizontal bars indicate the mean values for each type of cybrid. +*p* = 0.08 (Mann-Whitney *U* test).

The nucRP data from the first experiments indicated that the *8701A*/*10398A* cybrids tended to have higher peak cytosolic calcium levels after the histamine stimulation compared with the *8701G*/*10398G* cybrids (*8701A*/*10398A,* 2.10 ± 0.14 [mean ± SD], 11 cell lines; *8701G*/*10398G,* 2.02 ± 0.15, 22 cell lines, [mean ± SD]; *p* = 0.08, Mann-Whitney *U* test; Figure 4). There was no significant difference of basal cytosolic calcium level (*8701A*/*10398A,* 1.29 ± 0.07 [mean ± SD], 11 cell lines; *8701G*/*10398G,* 1.25 ± 0.07 [mean ± SD], 22 cell lines; *p* > 0.10, Mann-Whitney *U* test). This result suggests that cytosolic calcium response may be enhanced in the *8701A*/*10398A* cybrids.

The nucRP data from the replication study using cybrid cell lines stably expressing RP confirmed that peak cytosolic calcium levels after histamine stimulation were higher in the *8701A*/*10398A* cybrids than in *8701G*/*10398G* cybrids (*8701A*/*10398A,* 2.56 ± 0.03 [mean ± SD], three cell lines; *8701G*/*10398G,* 2.26 ± 0.16 [mean ± SD], three cell lines).

## Discussion

In the present study, we comprehensively searched for mtDNA polymorphisms that alter the fluorescence ratios of mtRP, and we identified two mtDNA single nucleotide polymorphisms (mtSNPs), *10398A* and *8701A,* that increase the basal fluorescence ratios of mtRP.

In the *10398A*/*G* mtSNP, threonine is substituted by alanine at the C terminus of ND3, a subunit of complex I (NADH: ubiquinone oxidoreductase) [10]. In the *8701A*/*G* mtSNP, there is an amino acid substitution from threonine to alanine at ATPase6, the F0 subunit 6 of complex V (ATP synthase) [10]. Complex I generates a proton gradient across mitochondrial inner membrane by exporting protons, whereas ATP synthase produces ATP by using this proton gradient. The proton gradient is also the driving force of calcium uptake by mitochondria. Therefore, both polymorphisms can affect the proton gradient across mitochondrial inner membrane and calcium levels.

During the course of our study, it was reported that the fluorescence ratio of RP is dependent not only on calcium levels but also on pH at the higher pH ranges in mitochondrial matrix [32,33]. Thus, we measured basal mitochondrial matrix pH in cybrids with *8701A*/*10398A* and *8701G*/*10398G.* Basal mitochondrial matrix pH was significantly lower in cybrids with *8701A*/*10398A* than in those with *8701G*/*10398G.* Alteration in mitochondrial pH has also been hypothesized to modulate ATP production, apoptosis, and the opening of the mitochondrial membrane permeability transition pore [44]. Transport of protons is coupled with electron transport by a respiratory chain consisting of complexes I, III, and IV. As described above, the mtDNA 10398 polymorphism affects the amino acid composition in a subunit of complex I. When the activity of complex I in the electron-transport chain was measured using citrate synthase activity as a reference [45], the activity of *8701A*/*10398A* cybrids tended to be lower than that of *8701G*/*10398G* cybrids (*8701A*/*10398A,* 5.77 ± 1.41 [mean ± SD], four cell lines; *8701G*/*10398G,* 7.58 ± 1.27 [mean ± SD], four cell lines; *p* = 0.10, *t* test). This finding supports the above-mentioned difference in mitochondrial matrix pH.

The difference in basal mitochondrial matrix pH could not contribute to the initial finding of higher mtRP fluorescence ratio in the cybrids with *8701A*/*10398A* because lower pH could only decrease the mtRP fluorescence rate, not increase it. Therefore, the most probable interpretation for the difference in basal mtRP fluorescence ratio is higher mitochondrial calcium levels in cybrids carrying *8701A*/*10398A.* In addition, the calcium responses to histamine stimulation tended to be higher in the cytosol of *8701A*/*10398A* cybrids than *8701G*/*10398G* cybrids, both in the initial experiment and the replication study using cybrid cell lines stably expressing RP. These findings suggest that alterations in mitochondrial function cause the enhanced cytosolic calcium response and higher basal mitochondrial calcium levels in cybrids with *8701A*/*10398A.*

Mitochondria have several calcium transport mechanisms. Calcium uptake is attributable to a mitochondrial calcium uniporter in inner mitochondrial membrane. Calcium efflux from mitochondria is mediated by the Na^+^/Ca^2+^ exchanger and the H^+^/Ca^2+^ antiporter. The opening of the membrane permeability transition pore also allows efflux of calcium from mitochondria. Clarification of mitochondrial calcium transport systems at the molecular level would facilitate the understanding of biochemical mechanism of how the 10398 polymorphism alters mitochondrial calcium dynamics. Further study is needed to reveal how these two mtSNPs alter mitochondrial matrix pH and calcium levels.

Among the two polymorphisms we found to have functional significance, *10398A*/*G* has previously been associated with human health and diseases. The *10398G* polymorphism is reportedly associated with a reduced risk of Parkinson disease [19], whereas *10398A* was reported to increase the risk of Alzheimer disease in men [17], invasive breast cancer in African-American women [24], and prostate cancer in African-American men [25]. In addition, *10398A* is reportedly associated with an increased risk of bipolar disorder [22,23,46]. According to the mtSNP database (http://www.giib.or.jp/mtsnp/index_e.html), most Europeans have *8701A,* but it is polymorphic in the Japanese population. In contrast, *10398A* is polymorphic both in Europeans and Japanese. The *10398A*/*G* characterizes the European haplogroup I, J, and K [17], and Asian-specific super haplogroup M [47]. Thus, the present findings can be translated that Asian-specific super haplogroup M is associated with different mitochondrial pH and calcium from other Asian-specific haplogroups.

The frequency of *10398A* is much smaller in the Japanese population (approximately 29% in the mtSNP database) compared with Europeans (approximately 74% in the mtSNP database). The average life expectancy in Japan is the highest in the world, and its biological basis is not yet well understood. Tanaka et al. sequenced the whole mtDNA sequence in 11 Japanese centenarians and reported that a subset of haplogroup M characterized by several mtDNA polymorphisms is more frequently seen in centenarians [48]. Among these polymorphisms, they have mainly focused on the *5178A* polymorphism that characterizes haplogroup D in super haplogroup M. The *5178C/A* is associated with several common diseases such as Parkinson disease [28], atherosclerosis in patients with diabetes mellitus [49], and myocardial infarction [50]. A recent study, however, suggested that association of *5178A* with longevity is ethnicity dependent [51]. Therefore, it might be possible that these apparent effects of *5178C*/*A* in the Japanese population may be mediated by the other linked polymorphisms that cause functional change. Although *5178A* did not affect the fluorescence ratio of mtRP in this study, most subjects with *5178A* have *10398G.* Thus, it could be possible that *10398G* mediates these apparent associations of *5178C*/*A* with complex diseases and longevity. In fact, based on the mtSNP database, *10398G* was significantly more frequently seen in centenarians (76 of 96 [79%]) compared with young Japanese (129 of 192 [67%]; *p* = 0.03). A recent study by Niemi et al. also suggested that *10398G* affects longevity [52].

To date, there has been no study on the association of *8701A*/*G* with health and disease, and information on *8701A*/*G* is limited. Most of the above-noted associations with the 5178 polymorphism observed in the Japanese population could also be mediated by 8701, since it is closely linked with 10398 and 5178. In fact, *8701G* is also more frequency seen in centenarians (68 of 96 [71%]) compared with young Japanese (111 of 192 [58%]; *p* = 0.03).

It was difficult to assess the functional effects of these two mtSNPs separately, because there were only two subjects carrying *8701A*/*10398G* in our sample set. Although it is still an open question whether or not *8701A*/*G* causes some functional change, at least *10398A*/*G* seems to have some functional significance. The tendency of altered complex I activity between the *8701A*/*10398A* and *8701G*/*10398G* cybrids suggest that the observed difference between *8701A*/*10398A* and *8701G*/*10398G* is mediated at least partly by the functional alteration of complex I by *10398A*/G*.*

Because there was considerable overlap, including an outlier, with regard to the basal mtRP fluorescence between *8701A*/*10398A* and *8701G*/*10398G,* there might be a concern that the observed difference may be caused by those outliers. Thus, we applied the Smirnov-Grubbs test for the detection of outliers. One cell line, having the lowest value in the *8701G*/*10398G* group, was found to be an outlier (T = 3.17; *p* < 0.05). Even after this cell line was omitted, basal mtRP fluorescence ratios in cybrids with *8701A*/*10398A* were significantly higher than those with *8701G*/*10398G* (*8701A*/*10398A,* 1.67 ± 0.09 [mean ± SD], *n* = 11; *8701G*/*10398G,* 1.60 ± 0.09 [mean ± SD], *n* = 21; *p* < 0.05, Mann-Whitney *U* test). Thus, the observed difference was not due to the effect of the outlier. One might suspect that observed variation of basal mtRP fluorescence ratios might not be caused by mtDNA sequence variation but resulted from an experimental variation. However, the interassay variance within one cybrid cell line having the same mtDNA sequence was significantly smaller than the variation among cybrid cell lines having different mtDNA sequences. This indicates that observed variation of mtRP fluorescence ratios among cybrid cell lines were not caused by experimental variation but caused by the difference of mtDNA sequence.

There might be the other concern that the present finding is somehow affected by the process of subcloning, and such a finding cannot be generalized. However, in our replication study using independently established cybrid cell lines, it was confirmed that the observed finding was not specific to this particular subcloned cybrids, but was actually caused by differences of mtDNA sequences.

In summary, we identified two mtSNPs that were suggested to alter mitochondrial matrix pH and intracellular calcium dynamics. To our knowledge, this is the first report of mtDNA polymorphisms affecting these intracellular functions. Among the two mtSNPs examined, the *10398A*/*G* polymorphism was previously reported to be associated with Parkinson disease, Alzheimer disease, some kinds of cancer, bipolar disorder, and longevity. A drug that affects mitochondrial matrix pH or calcium levels might be a promising strategy for health and disease.

## Materials and Methods

### Subjects.

Cybrids were generated from platelets derived from 35 volunteers, 17 patients with bipolar disorder (mean age, 48 years; 9 men and 8 women) and 18 healthy controls (mean age, 48 years; 10 men and 8 women). All were Japanese. Patients with bipolar disorder were included in this study because these blood samples were collected as a part of a project to study the genetics of bipolar disorder. None of the experimental parameters reported in this paper showed a significant difference between patients and controls. Diagnoses were made by the consensus of two senior psychiatrists using the DSM-IV criteria. Written informed consent was obtained from all volunteers. The Ethics Committees of the RIKEN Brain Science Institute and other participating institutes approved the study. For the replication study using cybrid cell lines stably expressing RP, we selected cybrids from three subjects with *8701A*/*10398A* mtDNA (mean age, 48 years; 1 man and 2 women) and three subjects with *8701G*/*10398G* mtDNA (mean age, 51 years; 1 man and 2 women). Cybrids for replication studies using independently established cybrid cell lines and measurements of mitochondrial matrix pH were generated using platelets from four *8701A*/*10398A* subjects (mean age, 52 years; 2 men and 2 women) and four *8701G*/*10398G* subjects (mean age, 56 years; 2 men and 2 women). Cybrids for replication studies and the measurement of mitochondrial matrix pH were derived from healthy controls. Although these cell lines have several mtDNA polymorphisms in addition to 8701 and 10398, they were carefully selected so that they have minimum difference in other positions of mtDNA.

### Generation of the 143B.TK^−^ ρ^0^206 cell line stably carrying calcium-sensitive probes.

The 143B.TK^−^ ρ^0^206 cell line derived from the osteosarcoma cell line (143B.TK^−^) was established by Attardi and King [30]. The 143B.TK^−^ ρ^0^206 cells were grown in DMEM (Invitrogen, Carlsbad, California, United States), supplemented with 10% FBS, 50 U penicillin, 50 μg/ml streptomycin, and 50 μg/ml uridine. The cells were grown on dishes 35 mm in diameter to 50% confluence. The cells were cotransfected with Lipofect AMINE PLUS (Invitrogen) with *ratiometric Pericam-nuc* (0.25 μg DNA/dish) and *ratiometric coxIV-Pericam* (0.75 μg DNA/dish) cDNAs. After 2 d, these cells were replated onto coverslips 14 mm in diameter and allowed to grow to 2.5% confluence with selection medium, supplemented with 10% FBS, 1,000 μg/ml geneticin (Invitrogen), and 50 μg/ml uridine. After 2 wk, a single colony with fluorescence in nuclei and mitochondria was picked up under a fluorescent microscope. mtDNA-specific PCR and Southern blot analysis were used to confirm that the cells lacked mtDNA. The cell lines were replated onto coverslips and stimulated by histamine (Sigma-Aldrich, Saint Louis, Missouri, United States) as described below. One of the ten cell lines had a reproducible calcium response without spontaneous calcium oscillation. This line was selected for further examination.

### Generation of cybrid cell lines.

Peripheral blood was drawn into a 10 ml Vactainer tube containing 1.5 ml acid citrate dextrose (BD Biosciences, Palo Alto, California, United States). Platelets were separated by centrifugation at a speed of 1,200 rpm for 20 min. The 143B.TK^−^ ρ^0^206 cells that had been stably expressing RPs were fused with platelets from individuals using a polyethylene glycol solution [53]. After 3 d of fusion, the medium was replaced by a selection medium without uridine. After 2 wk, surviving cells were collected. The integration of mtDNA was verified by Southern blot analysis as previously described with some modifications [54]. Cybrids for the replication study using independently established cybrid cell lines and measurements of mitochondrial matrix pH were generated by fusing the native 143B.TK^−^ ρ^0^206 cells and platelets from individuals as described above.

### Measurements of fluorescence ratios of RP.

Cybrids in modified Krebs R buffer (125 mM NaCl, 5 mM KCl, 5.5 mM glucose, 20 mM HEPES, and 1 mM MgCl_2_ [pH 7.4]) were imaged at room temperature on an Olympus IX-70 (Olympus, Tokyo, Japan) with a CoolSNAPHQ CCD camera (Roper Scientific, Tucson, Arizona, United States) controlled by Universal Imaging Meta series 4.5/4.6 (Universal Imaging, Media, Pennsylvania, United States). Dual-excitation imaging with RP used two excitation filters (480DF10 and 410DF10), alternated by a Lambda 10–2 filter exchanger (Sutter Instruments, Novato, California, United States), a 505DRLP-XR dichroic mirror, and a 525AF45 emission filter. The cybrids were stimulated by bath-application of 10 μM histamine applied to the coverslip. The ratio of the 525 nm fluorescence at 480 nm to that at 410 nm was used for further analysis. The regions of interest for measuring calcium response in each cell were the mitochondria and nucleus.

For each cell line, 11–42 cells were measured. At first, the basal fluorescence ratios of mtRP obtained from each cell were used for statistical analysis by one-way analysis of variance. Using one-way analysis of variance (*df* = 34), we tested whether or not the variation among 35 cybrid cell lines having different mtDNA was larger than the variation within one cell line. Then, the 11–42 data points in each cell line were averaged to calculate the representative value for each cell line.

The coefficient of variation in fluorescence ratio of mtRP in each cell line was 12.2% ± 3.2% (mean ± SD in 35 cybrids).

For the replication study using eight independently established cybrid cell lines (four with *8701A*/*10398A* mtDNA and four with *8701G*/*10398G* mtDNA), cybrids were transiently transfected with *ratiometric coxIV-Pericam* (1 μg DNA/dish) cDNA using Lipofect AMINE PLUS. After 2 d, measurements were performed in these cell lines. Different from the case in the cells stably expressing RP, there is no assurance that the expression level of RP is constant across cells in this experiment, which may potentially affect the stability of fluorescence ratio. To avoid the effect of variability of expression levels of RP among cells, we performed in vivo calibration for each cell. To exactly estimate calcium levels, we applied histamine stimulation to the cells, and only those responded to histamine were further analyzed. Cells responded to calcium could be found in three of four cell lines for each group. Cells were stimulated by 10 μM histamine, and calibration was performed using calibration buffers. Calcium-free buffer consisted of modified Krebs R buffer with 10 μM ionomycin (Calbiochem, San Diego, California, United States), 50 μM BAPTA-AM (Dojindo Laboratories, Kumamoto, Japan), and 10 mM EGTA (Dojindo Laboratories). Calcium saturation buffer was made of modified Krebs R buffer with 10 μM ionomycin and 10 mM CaCl_2_. [Ca^2+^] measured by in situ calibration was calculated by the equation [Ca^2+^] = *K′*_d_ [*(R* − *R*_min_ *)* / *(R*_max_ − *R)*]^(1/*n*)^, where *K′*_d_ is the apparent dissociation constant corresponding to the calcium concentration and *n* is the Hill coefficient [55]. Because *K′*_d_ and *n* are constants but *K′*_d_ of RP in mitochondria is not known, we simply used the value of *(R* − *R*_min_ *)* / *(R*_max_ − *R)* for the assessment of mitochondrial calcium level. The condition for imaging is basically similar to those described above.

### Analysis of entire sequences of mtDNA.

Total DNA was extracted from each cybrid using standard protocols. The entire mtDNA was sequenced as described [56]. A computer program, Sequencher version 4.1.1 (Gene Codes, Ann Arbor, Michigan, United States), was used to indicate possible SNP loci. For verification, visual inspection of each candidate SNP was carried out. At least two overlapping DNA templates amplified with different primer pairs were used for identification of each SNP. mtSNPs were identified by comparison with the revised Cambridge sequence (GI1944628) reported by Andrews et al. [37]. For all polymorphisms that resulted in changed amino acid sequence or those within rRNA or tRNA regions, averaged fluorescence ratios of mtRP were compared between genotypes by Mann-Whitney *U* test. This analysis was performed only when three or more cybrids had the same polymorphism. For statistical analysis, Mann-Whitney *U* tests were applied using SPSS software (SPSS, Tokyo, Japan).

### Construction of pH indicator for measurements of mitochondrial matrix pH.

*GFP* variants were prepared as described [57]. As a template, the original *GFP* mutation was employed. This template incorporates the mutation [38,58]. Mutations were verified by sequencing the entire gene. The *pH-GFP* [39] with polyhistidine tag at the N terminus was expressed in Escherichia coli JM109 (DE3) (Promega, Madison, Wisconsin, United States), purified, and spectroscopically characterized as described [55]. The gene for *mt pH-GFP* was amplified by PCR using the *GFP* variants as a template, with a forward primer containing *complex IV* mitochondrial target sequences and an MluI site, and a reverse primer containing an EcoRV site. The gene for *mt DsRed* was amplified by PCR using p*DsRed1–1 Mito* as a template with a forward primer containing a NotI site and a reverse primer containing a SalI site. The restricted *mt pH-GFP* product was subcloned in-frame into multiple cloning site I, and *mt DsRed* was subcloned into multiple cloning site II of the pBI bidirectional Tet vector (Clontech Laboratories, Mountain View, California, United States).

### Transient expression of pH indicator in cybrid cell lines using the Tet system.

To ensure the same expression levels of two genes, *mt pH-GFP* and *mt DsRed,* the bidirectional Tet expression vector was used in Tet-Off Gene expression systems [40,41]. The pBI bidirectional Tet expression vector contains a “bidirectional” promoter composed of a tetracycline-response element flanked by two minimal cytomegalovirus promoters in opposite orientations [42]. To induce these two genes, the other vector expressing the tetracycline-controlled transactivator under the cytomegalovirus promoter control was cotransfected.

Cybrids were grown in DMEM (Sigma-Aldrich) supplemented with 10% Tet system-approved FBS (Clontech), 100 U penicillin, and 100 μg/ml streptomycin. The cells were grown on dishes 35 mm in diameter to about 80% confluence. The cells were cotransfected with Tet-Off vector (Clontech) and pBI–*(mt pH-GFP)–(mt DsRed)* (total 1 μg DNA/dish) using Lipofect AMINE PLUS (Invitrogen). After 2 d, mitochondrial matrix pH was measured in these cells.

### Mitochondrial matrix pH measurements and in vivo calibration.

Cybrids in modified Krebs R buffer were imaged at room temperature. The imaging system is described above. Imaging with mt pH-GFP/DsRed used two excitation filters (480DF10 and S565/25), a dichroic mirror for GFP/DsRed, and emission filters (525AF45 and S620/60).

It was confirmed that the fluorescence ratio (525 nm/620 nm) decreased after the treatment with FCCP (Sigma-Aldrich), suggesting that this set of fluorescent indicators is sensitive to the mitochondrial matrix pH.

For in situ calibration, cybrids were perfused with the following pH titration buffer (125 mM KCl, 20 mM NaCl, 0.5mM CaCl_2_, 0.5 mM MgCl_2_, and 25 mM pH buffer [MOPS was used for pH 7.0, HEPES for pH 7.5 and 8.0, and glycylglycine for pH 8.5]) [59]. All calibration experiments were carried out in the presence of 10 μM nigericin and 10 μM monensin (Sigma-Aldrich). After baseline measurements were recorded for several minutes, calibration was performed in vivo. The fluorescence ratio of 525 nm emission at 480 nm excitation to 620 nm emission at 565 nm excitation reflected the dynamics of mitochondrial matrix pH. Mitochondrial matrix pH was calculated by plotting the calibration curve of each cell.

## Supporting Information

Figure S1 Effects of *8701A*/*10398A* and *8701G*/*10398G* on Basal Mitochondrial Calcium Levels Measured by mtRP The *y* axis shows the basal mitochondrial calcium levels indicated by the value of *(R* − *R*_min_ *)* / *(R*_max_ − *R)* measured by mtRP. In situ calibration for [Ca^2+^] uses the equation [Ca^2+^] = *K′*_d_ [*(R* − *R*_min_ *)* / *(R*_max_ − *R)*]^(1/*n*)^. *R* is the basal ratio of the 525 nm fluorescence at 480 nm to that at 410 nm. *R*_min_ is the ratio at free calcium. *R*_max_ is the ratio at saturating calcium. *K′*_d_ is the apparent dissociation constant. *n* is the Hill coefficient. Bars indicate the standard error of mean for each cybrid cell line.
(120 KB PDF)

Table S1 mtDNA Polymorphisms in Eight Cybrids (97 KB XLS)

## Abbreviations

cybrid: transmitochondrial hybrid cell
mt DsRed: mitochondria-targeted red fluorescent protein from Discosoma
mt pH-GFP: mitochondria-targeted pH-sensitive green fluorescent protein
mtDNA: mitochondrial DNA
mtRP: ratiometric Pericam targeted to mitochondria
mtSNPs: mitochondrial DNA single nucleotide polymorphisms
nucRP: ratiometric Pericam targeted to nucleus
RP: ratiometric Pericam
rRNA: ribosomal RNA
tRNA: transfer RNA
