# Supplementary material for: Identification of Mitochondrial DNA Polymorphisms That Alter Mitochondrial Matrix pH and Intracellular Calcium Dynamics
Source: PLoS Genet. 2006 Aug 11;2(8):e128. doi: 10.1371/journal.pgen.0020128 (PMC1534079; doi:10.1371/journal.pgen.0020128)
Supplement: Supplementary file 1 [file pgen.0020128.sg001.pdf]

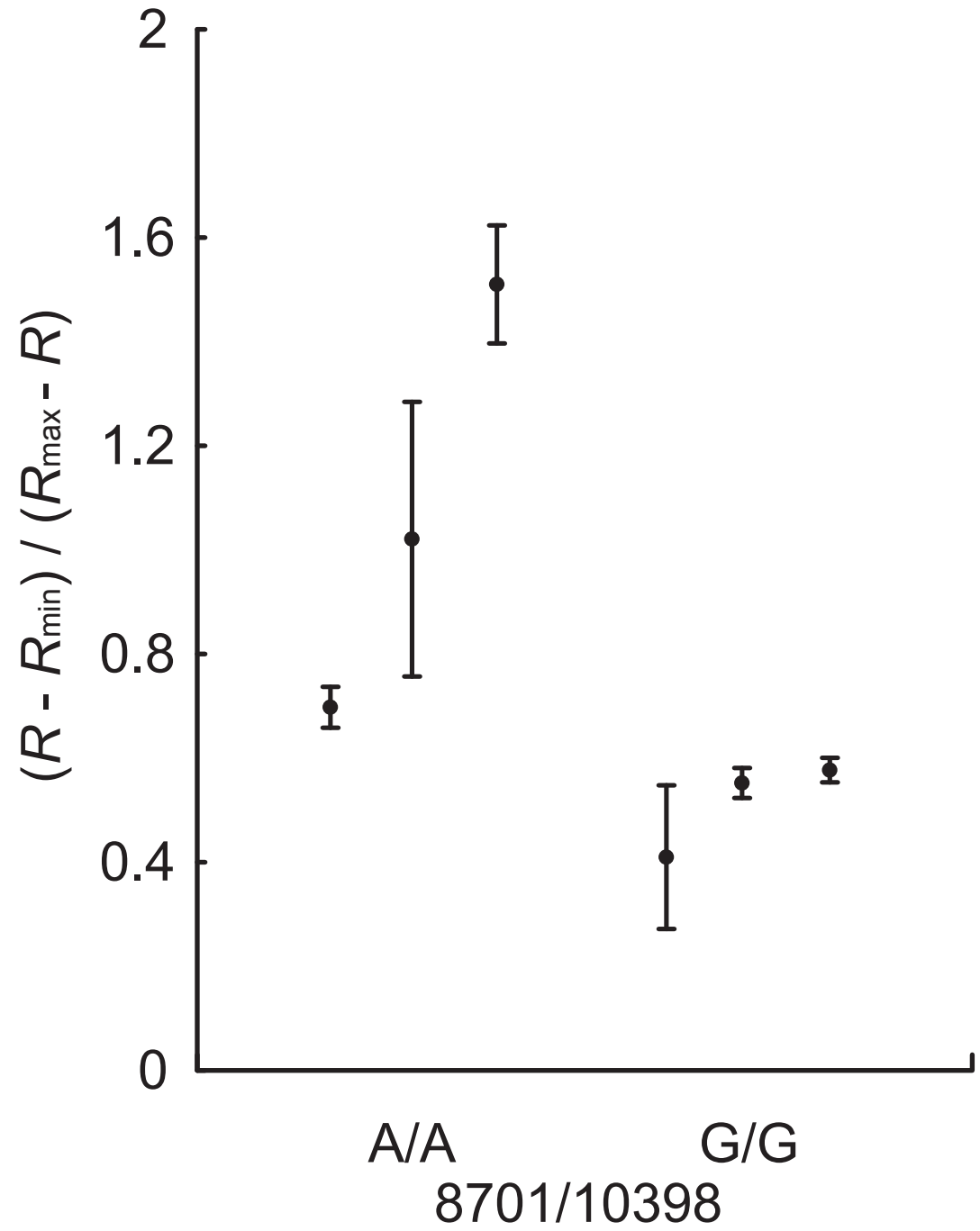
2
1.6
1.2
0.8
0.4
0
$(R - R_{min}) / (R_{max} - R)$
A/A
G/G
8701/10398
